# Oxidative lesions modulate G-quadruplex stability and structure in the human BCL2 promoter

**DOI:** 10.1093/nar/gkab057

**Published:** 2021-02-08

**Authors:** Stasė Bielskutė, Janez Plavec, Peter Podbevšek

**Affiliations:** Slovenian NMR Center, National Institute of Chemistry, Hajdrihova 19, SI-1000 Ljubljana, Slovenia; Slovenian NMR Center, National Institute of Chemistry, Hajdrihova 19, SI-1000 Ljubljana, Slovenia; EN-FIST Center of Excellence, Trg OF 13, SI-1000 Ljubljana, Slovenia; Faculty of Chemistry and Chemical Technology, University of Ljubljana, Večna pot 113, SI-1000 Ljubljana, Slovenia; Slovenian NMR Center, National Institute of Chemistry, Hajdrihova 19, SI-1000 Ljubljana, Slovenia

## Abstract

Misregulation of *BCL2* expression has been observed with many diseases and is associated with cellular exposure to reactive oxygen species. A region upstream of the P1 promoter in the human *BCL2* gene plays a major role in regulating transcription. This G/C-rich region is highly polymorphic and capable of forming G-quadruplex structures. Herein we report that an oxidative event simulated with an 8-oxo-7,8-dihydroguanine (^oxo^G) substitution within a long G-tract results in a reduction of structural polymorphism. Surprisingly, ^oxo^G within a 25-nt construct boosts thermal stability of the resulting G-quadruplex. This is achieved by distinct hydrogen bonding properties of ^oxo^G, which facilitate formation of an antiparallel basket-type G-quadruplex with a three G-quartet core and a G·^oxo^G·C base triad. While ^oxo^G has previously been considered detrimental for G-quadruplex formation, its stabilizing effect within a promoter described in this study suggests a potential novel regulatory role of oxidative stress in general and specifically in *BCL2* gene transcription.

## Graphical Abstract

**Graphical Abstract:**
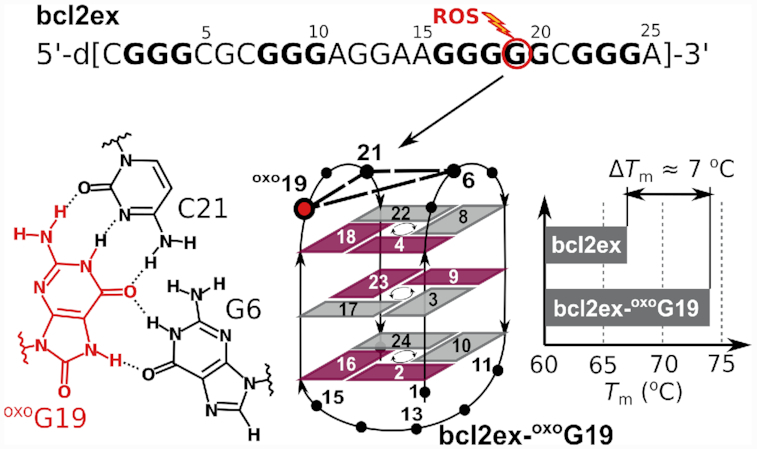
^oxo^G influences the fold of a G-quadruplex structure within the human BCL2 promoter and enhances the thermal stability of the G4 structure by forming novel structural elements.

## INTRODUCTION

Apoptosis or programmed cell death is essential in eliminating damaged and diseased cells (1). The BCL2 family of proteins contains several structurally and functionally related members, which are important regulators of apoptosis found primarily on the outer surface of mitochondrial membranes. Some proteins, like BCL2, have anti-apoptotic effects while others are pro-apoptotic. The balance between pro- and anti-apoptotic proteins regulates cell survival (2). Excessive apoptosis was found problematic with non-regenerative tissues and can lead to neurodegenerative diseases (3). BCL2 overexpression, on the other hand, tilts the balance in favour of cell survival. The *BCL2* gene was found to be aberrantly overexpressed in a wide range of human tumours, including B-cell and T-cell lymphomas, breast, lung, prostate, cervical and colorectal cancers as well as gliomas, melanomas and neuroplastomas (4). Furthermore, as an essential survival factor, BCL2 was found to protect cells experiencing spikes in oxidative stress from apoptosis (5,6). While lacking redox domains, its anti-oxidant effects were suggested to be indirect, involving modulation of mitochondrial bioenergetics (7).

DNA sequences with tandem G repeats can fold into four-stranded structures called G-quadruplexes (8). Promoters of tightly regulated genes related to tumorigenesis often contain guanine (G) rich regions suggesting their role in transcriptional control (9). The human *BCL2* gene has two promoter regions, P1 and P2 (10). The major promoter P1 is G/C-rich, contains multiple transcription start sites and was shown to play a major role in regulating *BCL2* transcription (11). *BCL2* overexpression in a wide range of human tumours made G-quadruplex structures within its gene promoter attractive targets for cancer therapeutics. Promising results in downregulating the gene were obtained by stabilizing non-canonical secondary structures within its promoter with organometallic complexes and heterocyclic ligands such as quindoline, perylene, coronene and naphthelene-diimide derivatives (12).

Generation of reactive oxygen species (ROS) is a part of normal cellular metabolism and exerts a constant strain on DNA. Among the four DNA nucleobases, guanine has the lowest redox potential and is therefore the easiest to oxidize (13,14). Based on theoretical studies, successive runs of Gs are even more susceptible to oxidation than isolated nucleotides (15). Numerous nucleotide analogues resulting from guanine oxidation have been reported thus far of which 8-oxo-7,8-dihydroguanine (^oxo^G) is commonly used as a biomarker for oxidative stress (16,17). ^oxo^G nucleotides prefer the *syn* conformation supposedly due to reduced repulsions between O8 and O4’ of the sugar (18). Furthermore, hydrogen bonding properties of ^oxo^G differ from the parent nucleotide as the Hoogsteen edge contains the protonated N7, which is a hydrogen bond donor in contrast to G where N7 is an acceptor. Guanine oxidation sites were found to be enriched in open chromatin regions and regulatory elements (19). In coding regions ^oxo^G and its derivatives cause mutations and transcription stalling (20). In promoter regions ^oxo^G was suggested to regulate transcription through changing structural properties and stability of G-quadruplex DNA (21,22). It is commonly and mistakenly thought that ^oxo^G substitutions are detrimental for G-quadruplex stability and structure (23–25). ^oxo^G was found to exhibit a stabilizing effect when substituted to outer G-quartets of tetramolecular G-quadruplexes (26).

**Figure 1.**
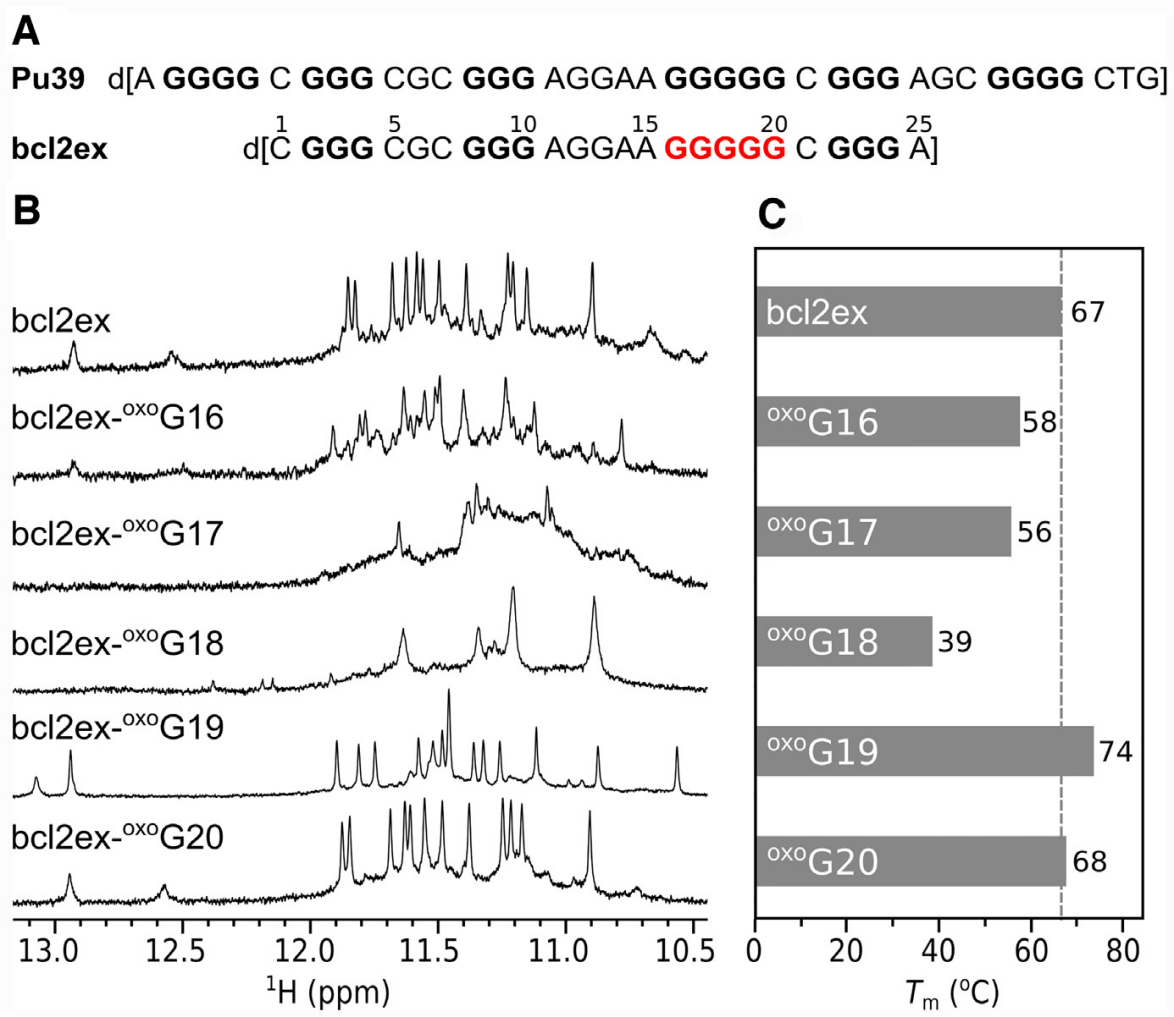
(**A**) DNA oligonucleotides derived from the P1 promoter region of the *BCL2* gene. Nucleotides of the G_5_-tract, which were chosen for ^oxo^G substitutions are in red. (**B**) Imino regions of 1D ^1^H NMR spectra and (**C**) melting temperatures of bcl2ex and its analogues.

A 39-nt region within the P1 promoter of the *BCL2* gene with the highest G/C frequency was designated as Pu39 (Figure 1A) (27). Its sequence contains six runs of three or more consecutive Gs separated by two or more nucleotides. Four central G-tracts of Pu39 were shown to form stable G-quadruplexes; however, these also contain the G_5_-tract comprised of five consecutive guanines, which is a source of structural polymorphism (28). We designed a 25-nt construct (bcl2ex) containing the four central G-tracts with 5′ and 3′ single nucleotide overhangs (Figure 1A). All, except 3′, guanines in G-tracts are comparably sensitive to oxidation (29,30). However, three G_3_-tracts in bcl2ex possess no redundant Gs and substituting them with ^oxo^G would likely prevent the formation of a G-quadruplex structure. In genomic DNA oxidation of shorter G_3_-tracts would likely exclude the damaged G-tracts from G-quadruplex formation altogether. Instead, one of the two extra G-tracts would step in as a ‘spare tire’ (29,31). On the other hand, the G_5_-tract contains two redundant Gs and we set out to evaluate the (de)stabilizing effect of ^oxo^G substitutions within the G_5_-tract and establish potential changes in which nucleotides are involved in G-quartet formation and stacking. Only individual substitutions were tested as any successive oxidative events would take place on the initially oxidized ^oxo^G nucleotide due to its lowest redox potential (32). Our initial hypothesis stipulates that due to its distinct hydrogen bonding properties, ^oxo^G is excluded from G-quartet formation in bcl2ex via strand slippage. Furthermore, structural changes may have a negative effect on the stability of G-quadruplexes and the degree of destabilization depends on the position of individual substitution of ^oxo^G within the G_5_-tract of bcl2ex. Since single substitutions at all positions except the central one retain at least three consecutive Gs we did not expect a change in G-quadruplex topology. On the other hand, substitution in the middle formally splits the G_5_-tract rendering only two pairs of consecutive Gs available for G-quartet formation, which is expected to substantially reduce G-quadruplex stability. Surprisingly, our data show that a substitution with ^oxo^G at a distinct position within bcl2ex results in reduction of structural polymorphism and in an unanticipated increase in thermal stability. Furthermore, the parent (non-substituted) oligonucleotide adopts an antiparallel basket-type G-quadruplex structure, which expands the array of non-B-DNA structures associated with the *BCL2* promoter. Both bcl2ex and its analogue containing ^oxo^G exhibit novel structural elements, which deepen our understanding of G-quadruplex folding in general.

## MATERIALS AND METHODS

### Sample preparation

All oligonucleotides were synthesized on a K&A Laborgeraete DNA/RNA Synthesizer H-8 using standard phosphoramidite chemistry. Isotopically labelled samples contained 8% ^13^C,^15^N-enriched guanine, adenine and cytosine nucleotides. Oligonucleotides were deprotected with AMA (1:1 mixture of aqueous ammonium hydroxide and methylamine) at 65°C for 30 min. Samples were purified using reverse-phase HPLC chromatography, followed by removal of the DMT group with 80% AcOH for 30 min. Then the oligonucleotides were transferred to a pure water phase by ether extraction and desalted using FPLC and a Sephadex G25 column. DNA solutions were dried on a vacuum centrifuge and redissolved in 9:1 H_2_O/^2^H_2_O, 20 mM potassium phosphate buffer, pH 7, and 70 mM KCl. Then the samples were concentrated using Amicon Ultra – 0.5 ml Centrifuge Filters. Final NMR solutions collected from Amicon tubes were heated to 95°C for 10 min and left to cool on the bench. DNA concentrations were determined by measuring UV absorption at 260 nm. Extinction coefficients of 231 300, 251, 500 and 251 300 M^−1^cm^−1^ were calculated with the nearest-neighbour method for nonsubstituted bcl2MidG4, bcl2ex and bcl2exT, respectively. Also, the same extinction coefficients were used for oligonucleotides contained ^oxo^G substitution. The final oligonucleotide concentrations were in the range from 0.5 to 1.2 mM. bcl2ex and bcl2ex-^oxo^G19 samples for ^1^H–^2^H exchange experiment, were dried on a vacuum centrifuge and redissolved in ^2^H_2_O.

### UV melting and thermodynamic analysis

UV melting experiments were performed on an Agilent Cary 3500 UV–Vis spectrophotometer using 1 cm path length cells. Samples were heated/cooled at a rate of 0.5°C/min in the range of 10–95°C and absorbance at 295 nm was monitored. *T*_m_ were determined from the first derivative of *A*_295_ versus temperature plot.

### Circular dichroism (CD) spectroscopy

CD spectra were acquired on Chirascan CD spectrometer at 25°C using 2.0 mm light path quartz cuvettes. All samples for CD were prepared by dilution of NMR samples to final 50 μM DNA concentrations in 20 mM potassium phosphate buffer, pH 7, and 70 mM KCl solution. CD spectra were acquired in the wavelength range from 220 to 320 nm.

### NMR experiments

All NMR data were collected on Agilent/Varian 600 and 800 MHz NMR spectrometers at 25°C. All homonuclear spectra were acquired with DPFGSE solvent suppression. 2D NOESY experiments (*τ*_m_ = 80, 150, 200, 250, 300, 350 ms) were utilized for ^1^H resonance assignments. Identification of guanine imino proton resonances was aided by 1D ^15^N-edited HSQC experiments, while aromatic of adenine, guanine and cytosine proton resonances were assigned with the help of ^13^C-edited HSQC experiments using 8% ^13^C,^15^N-labeled oligonucleotides. 2D TOCSY (*τ*_m_ = 80 ms) and DQF-COSY experiments gave information on sugar puckering and cytosine H5 protons resonances. Also, 2D ^1^H–^13^C HSQC spectra were used to help to assign H8/H2 aromatic resonances of adenines, guanines and to confirm *syn* conformation of nucleotides. Data were analysed with VNMRJ, NMRPipe, CcpNMR and Origin (33,34).

### Distance and dihedral angle restrains

Interproton distances were calculated from 200 ms and 250 ms NOESY spectra of bcl2ex-^oxo^G19 and bcl2ex, respectively, which were chosen from NOE buildup curves. The average of cytosine H6-H5 proton distances was used as a reference (2.47 Å). Restraint bounds for cross-peaks in aromatic and anomeric regions were set at ±0.5 Å for *d*_*ij*_ ≤ 3 Å, ±0.6 Å for 3 Å < *d*_*ij*_ ≤ 4 Å, ±0.7 Å for 4 Å < *d*_*ij*_ ≤ 4.5 Å, and ±0.8 Å for *d*_*ij*_ > 4.5 Å. For some restraints corresponding to overlapping or broader cross-peaks, larger distance ranges were used (±0.9–1.5 Å). All cross-peaks in imino-aromatic, imino-imino, amino-imino, and amino-aromatic regions were classified as strong (2.5 Å), medium (3.5 Å) or weak (4.5 Å) with ±1–1.5 Å restraint ranges.

Dihedral angle *χ* was restrained to a range between -90° and 90° for *syn* and between 90° and 270° for *anti* nucleotides, and ^oxo^G19 was left unrestrained. Restraints for backbone dihedral angles included *α* (–120–120°), *β* (150–210°), *γ* (30–90°), *δ* (130–190°), *ϵ* (170–300°), and *ζ* (–120–120°). All sugar puckers were determined to be of S-type (*C2*′*-endo*) based on 2D TOCSY and DQF-COSY NMR spectra, expect ^oxo^G19, which was determined to adopt a predominate N-type (*C3*′*-endo*) sugar pucker.

### Structural calculations

Molecular dynamics calculations were performed with AMBER 18 software using the ff99bsc0 force field and *ϵ/ζ*OL1 and *χ*OL4 modifications (35,36). Force field parameters for ^oxo^G nucleotide were derived from the RESP ESP charge derive (RED) Server (37).

Calculations were started from initial linear structure of the oligonucleotide, created with the LEAP module of AMBER 18. A total of 100 structures were obtained in 100 ps restrained SA simulations using Born implicit solvent model with random starting velocities. In the first 20 ps of SA, the temperature was kept at 1000 K, followed by slow cooling in the next 60 ps down to 300 and to 0 K in the last 20 ps. Force constants were 50 kcal·mol^−1^Å^−2^ for hydrogen bonds, 20 kcal·mol^−1^Å^−2^ for NOE distance restrains, 200 kcal·mol^−1^rad^−2^ for backbone, and 50 kcal·mol^−1^rad^−2^ for the χ torsion angle. Twelve structures were selected based on the lowest energy and subjected to energy minimization with a maximum of 10000 steps.

## RESULTS

### ^oxo^G can reduce G-quadruplex polymorphism

A construct (bcl2ex) containing the central four G-tracts of Pu39 with 5′ and 3′ single nucleotide overhangs (C1 and A25) exhibits a ^1^H NMR spectrum with narrow imino resonances in the Hoogsteen chemical shift range (Figure 1) suggesting formation of a G-quadruplex structure in the presence of K^+^ ions (vide infra). Following this interesting observation we probed the ability of the bcl2ex structure to tolerate oxidative lesions by individually substituting guanine nucleotides in the G_5_-tract with ^oxo^G and examined G-quadruplex forming ability of bcl2ex analogues with ^1^H NMR and UV melting experiments (Supplementary Figure S1). ^oxo^G20 substitution at the 3′ terminus of the G_5_-tract resulted in only minor chemical shift changes of imino resonances and no reduction in *T*_m_ indicating that the G-quadruplex fold is not perturbed. At the 5′ terminus of the G_5_-tract, the ^oxo^G16 substitution resulted in a 9°C decrease in *T*_m_ and occurrence of multiple structures in solution. However, the major species of bcl2ex-^oxo^G16 did exhibit similar imino resonance chemical shifts as bcl2ex suggesting that some of the parent fold is retained. Further reduction in thermal stability as well as broad NMR resonances indicative of multiple structures and/or aggregation were observed for ^oxo^G17 and especially ^oxo^G18 substitutions. (Figure 1B). Interestingly, chemical shifts of imino resonances of bcl2ex-^oxo^G19 differed considerably from the parent bcl2ex suggesting substantial changes in G-quadruplex structure (Figure 1B). Surprisingly, a 7°C increase in *T*_m_ was observed indicating that an oxidative lesion can lead to stabilization of a G-quadruplex, which stimulated us to analyse the relevant structures in detail.

CD spectra of bcl2ex and bcl2ex-^oxo^G19 were acquired for an initial assessment of their structural properties. While the CD spectrum of bcl2ex exhibits a strong positive signal at 265 nm and a less intense one at 295 nm, spectral properties of bcl2ex-^oxo^G19 differ considerably with positive signals at 250, 270 and 295 nm (Supplementary Figure S2). Spectral discrepancies suggest large differences in base stacking geometry of bcl2ex and bcl2ex-^oxo^G19 G-quadruplexes.

### bcl2ex forms a unique antiparallel basket-type G-quadruplex with a CGAA-quartet

**Figure 2.**
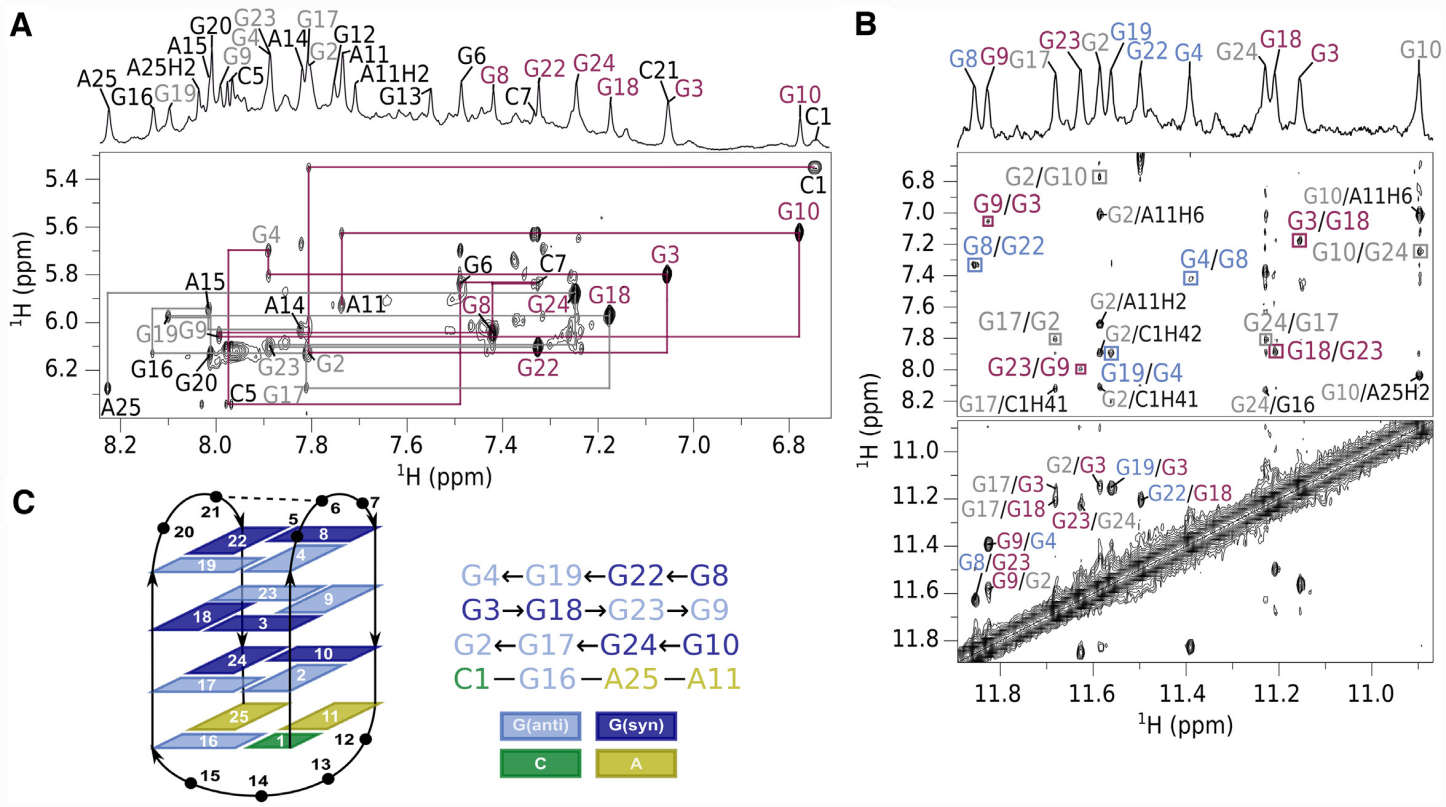
Assignment of bcl2ex spectra and G-quadruplex topology. (**A**) Aromatic-anomeric region of a NOESY spectrum (τ_m_ = 300 ms). Assignments are shown next to intranucleotide H6/H8-H1' cross-peaks. Magenta lines delineate the sequential walk from C1 to A11 and grey lines from A14 to A25. Resonances are labeled with extra-G-quartet nucleotides in black, while *syn* and *anti* guanines in G-quartets are in magenta and grey, respectively (**B**) Imino-imino and imino-aromatic regions of a NOESY spectrum. Cross-peaks in squares mark connectivities within G-quartets. (**C**) Topology of the bcl2ex G-quadruplex and hydrogen bond donor–acceptor directionalities. Hydrogen bonded nucleotides in loops are connected by dashed lines.

The ^1^H spectrum of bcl2ex exhibits 12 well-resolved guanine imino proton resonances in the Hoogsteen region from δ 10.90–11.85 ppm and two additional imino proton resonances in the Watson–Crick region at δ 12.52–12.93 ppm (Figure 1B). However, a broad background, especially in the aromatic region, and a set of low intensity signals suggest that minor species are also present (<10%). Imino and aromatic ^1^H resonances were unambiguously assigned using ^13^C and ^15^N isotopic enrichment (Supplementary Figure S3) and connectivities in 2D NOESY spectra (Figure 2). The G20T substitution (bcl2exT) offered superior spectral quality with virtually identical chemical shifts and confirmed spectral assignment of bcl2ex (Supplementary Figures S3 and S4). It is noteworthy that G for T substitutions in other positions of the G_5_-tract resulted broad resonances with perturbed chemical shifts. Seven intense H8/H6-H1’ cross-peaks could be observed in a NOESY spectrum of bcl2ex acquired with a mixing time of 80 ms. Six of them were assigned to guanines in *syn* conformation (G3, G8, G10, G18, G22 and G24). The remaining intense inter-nucleotide H6–H1’ NOESY cross-peak was assigned to C1. The *syn* conformation of C1 was confirmed by a downfield shift (δ 147.5 ppm) of its C6 resonance (Supplementary Figure S5). The bcl2ex sequence was incrementally extended in the 5′ direction with up to four Gs corresponding to the preceding G-tract from Pu39 to check if the *syn* conformation of C1 persists. However, additional Gs resulted in structural polymorphism and poor spectral quality precluding a detailed analysis (Supplementary Figure S6). Interestingly, the NMR spectrum of GG-bcl2ex was of sufficient quality to yield multiple imino resonances in the Hoogsteen region as well as two resonances in the Watson–Crick region suggesting its base pairing is in agreement with bcl2ex.

NOE connectivities in imino-imino and imino-aromatic regions were used to establish the fold of G-quadruplex structure, which is comprised of G2·G17·G24·G10, G3·G18·G23·G9 and G4·G19·G22·G8 quartets (Figure 2 and Supplementary Figure S7). Outer G-quartets exhibit *anti-anti-syn-syn* glycosidic conformations as well as counter-clockwise hydrogen-bond donor–acceptor directionalities. On the other hand, the inner G-quartet exhibits *syn–syn–anti–anti* glycosidic conformations and a clockwise hydrogen-bond directionality. G-tracts are connected by three loops of which the first (C5–G6–C7) and last (G20–C21) are of the edgewise type, while the longer second loop (A11–G12–G13–A14–A15–G16) is diagonal. Intra-nucleotide G12H8-H1’ and G13H8-H1’ cross-peaks could not be observed most likely due to the dynamic character of this part of loop region with its orientation changing on the intermediate NMR timescale. These structural features are consistent with the antiparallel basket-type G-quadruplex topology (Figure 2C). The two broader imino signals at δ 12.52 and 12.93 ppm (Figure 1B) were assigned to G6 and G16, respectively, and suggest their base pairing.

**Figure 3.**
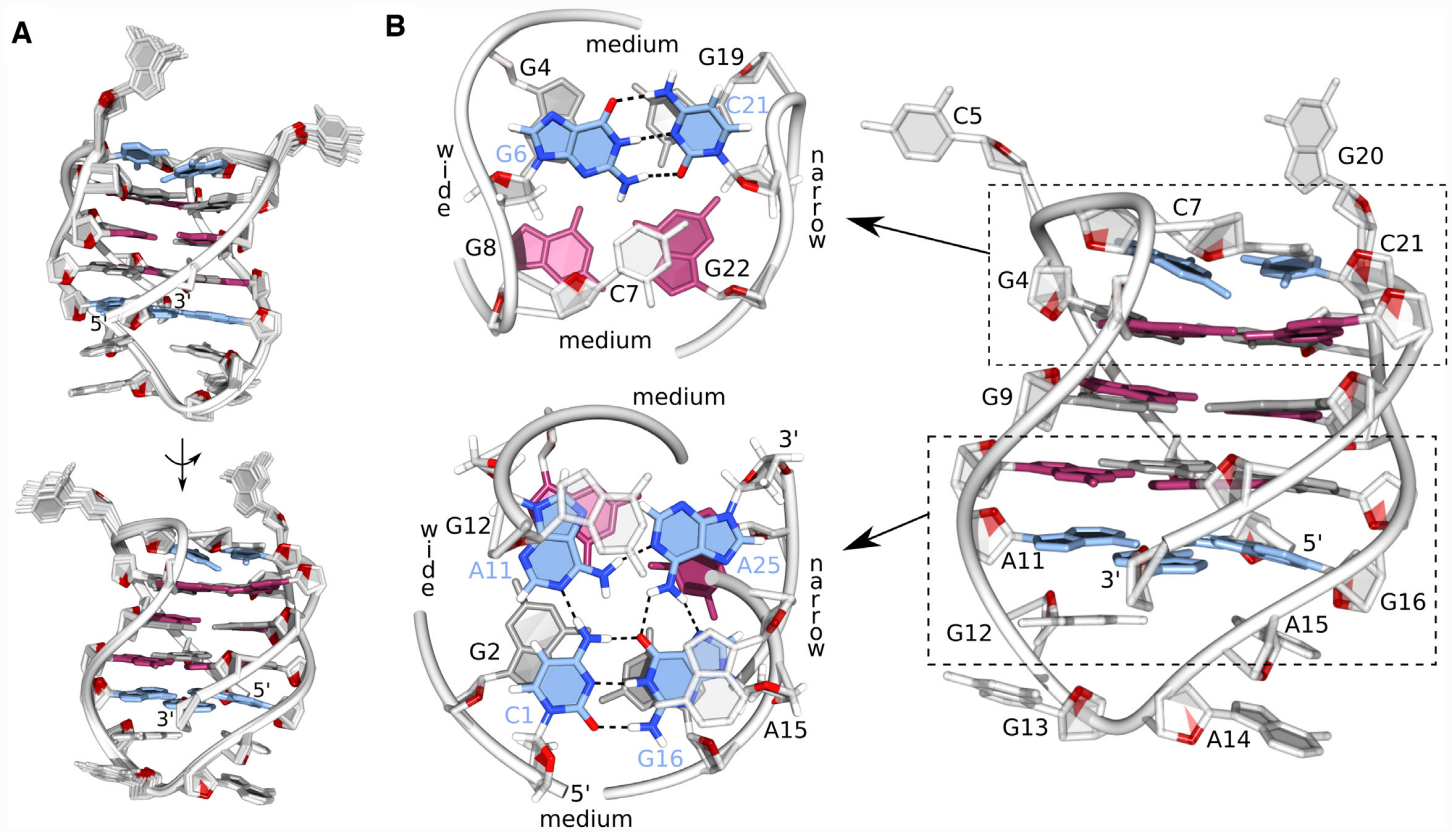
Structure of the bcl2ex G-quadruplex. (**A**) Superpositions of 12 lowest energy structures presented in two views rotated by 180°. (**B**) Details of the representative structure. *Syn* and *anti* guanines are shown in magenta and grey, respectively, base pairs and the GCAA-quartet are in blue.

A simulated annealing protocol was used to determine the high-resolution structure of the bcl2ex G-quadruplex. A good convergence of structures was achieved with on average more than 9 NOE distance restraints per nucleotide (Table 1 and Figure 3). The bcl2ex structure exhibits one wide groove (∼21 Å) between G2–G4 and G8–G10 tracts; two medium grooves (∼16 Å) between G2–G4 and G17–G19 tracts as well as between G8–G10 and G22–G24; and one narrow groove (∼9 Å) between G17–G19 and G22–G24 tracts (Figure 3B). Extra-G-quartet regions stabilize the G-quadruplex core with G6 and C21 forming a Watson–Crick base pair, which alongside C7 efficiently stack on the adjacent G-quartet. C5 and G20 are flexible and rotated away from the G-quadruplex core. On the opposite side of the structure, C1 and G16 form a Watson–Crick base pair, which interacts with A11 and A25 via hydrogen bonds to form a planar CGAA-quartet (Supplementary Figure S8). The diagonal loop is stabilized by stacking interactions between A14, A15 and G16 (Figure 3).

**Table 1.** Statistics of bcl2ex and bcl2ex-oxoG19 structures

	bcl2ex (6ZX7)	bcl2ex-^oxo^G19 (6ZX6)
NMR restraints		
Total NOE distance restraints	229	316
intranucleotide	133	168
internucleotide	96	148
sequential	55	83
long-range	41	65
Hydrogen-bond restraints	30	28
Hoogsteen	24	24
Watson–Crick	6	4
Glycosidic torsion angle restraints	12	13
Backbone torsion angle restraints	146	146
Deviations from idealized geometry		
Bond lengths (Å)	0.01	0.01
Torsion angles (°)	2.45	2.47
Pairwise heavy Atom RMSD (Å)		
G-quartets	0.16	0.15
All heavy atoms	0.28	0.86

### bcl2ex-^oxo^G19 adopts an antiparallel basket-type G-quadruplex structure stabilized by a base triad containing ^oxo^G

**Figure 4.**
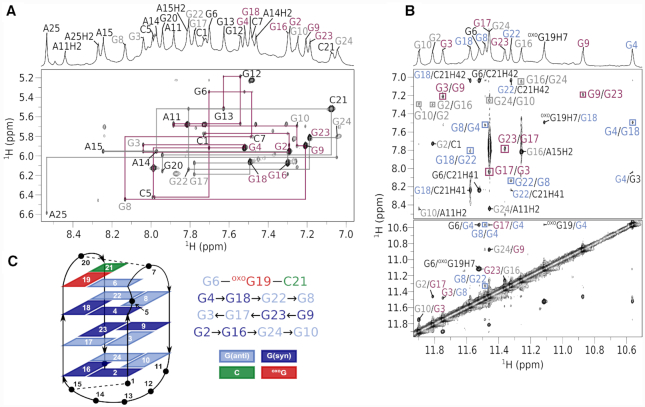
Assignment of bcl2ex-^oxo^G19 spectra and G-quadruplex topology (**A**) Aromatic-anomeric region of a NOESY spectrum (τ_m_ = 300 ms). Assignments are shown next to intranucleotide H6/H8–H1' cross-peaks. Magenta lines delineate the sequential walk from C1 to G13 and grey lines from A14 to A25. Resonances are labeled with extra-G-quartet nucleotides in black, while *syn* and *anti* guanines in G-quartets are in magenta and grey, respectively. (**B**) Imino-imino and imino-aromatic regions of a NOESY spectrum. Cross-peaks in squares mark connectivities within G-quartets. (**C**) Topology of the bcl2ex-^oxo^G19 G-quadruplex and hydrogen bond donor–acceptor directionalities. Hydrogen bonded nucleotides in loops are connected by dashed lines.

The ^1^H NMR spectrum of bcl2ex-^oxo^G19 exhibits 13 resolved imino resonances in the Hoogsteen region from δ 10.57 to 11.90 ppm and two additional signals in the Watson–Crick region at δ 12.94 and 13.07 ppm (Figure 1B). Imino and aromatic ^1^H resonances were unambiguously assigned using ^13^C and ^15^N isotopic enrichment (Supplementary Figure S9) and connectivities in NOESY spectra (Figure 4). Intense H8-H1' cross-peaks in a NOESY spectrum with a short mixing time of 80 ms were identified for G2, G4, G9, G16, G18, G23 and C21, which were assigned the *syn* conformation along their glycosidic bonds. Although the conformation of ^oxo^G19 could not be probed by analysing intensity of the H8–H1’ NOESY cross-peak, its H2’ and H2’ resonances were found at δ 2.98 and 2.68 ppm, respectively, which is downfield compared to *anti* guanines and suggests ^oxo^G19 is in *syn*. Twelve Gs involved in Hoogsteen hydrogen bonding were found to constitute the core of the bcl2ex-^oxo^G19 G-quadruplex, which is comprised of G2·G16·G24·G10, G3·G17·G23·G9 and G4·G18·G22·G8 quartets (Figure 4C). The outer G-quartets exhibit *syn–syn–anti–anti* glycosidic conformations as well as clockwise hydrogen bond donor/acceptor directionalities. On the other hand, the inner G-quartet exhibits *anti–anti–syn–syn* conformations and a counter-clockwise hydrogen bond directionality. G-tracts were found to be connected by three loops of which the first (C5–G6–C7) and last (^oxo^G19–G20–C21) are of the edgewise type, while the second one (A11–G12–G13–A14–A15) is a diagonal loop. The structure of bcl2ex-^oxo^G19 is consistent with an antiparallel basket-type G-quadruplex topology (Figure 4C). Additionally, three extra-G-quartet nucleotides exhibit observable imino signals in the ^1^H NMR spectrum of bcl2ex-^oxo^G19; G6H1 and ^oxo^G19H7 resonate in the Hoogsteen region and ^oxo^G19H1 and G20H1 in the Watson–Crick region (Supplementary Figure S9).

**Figure 5.**
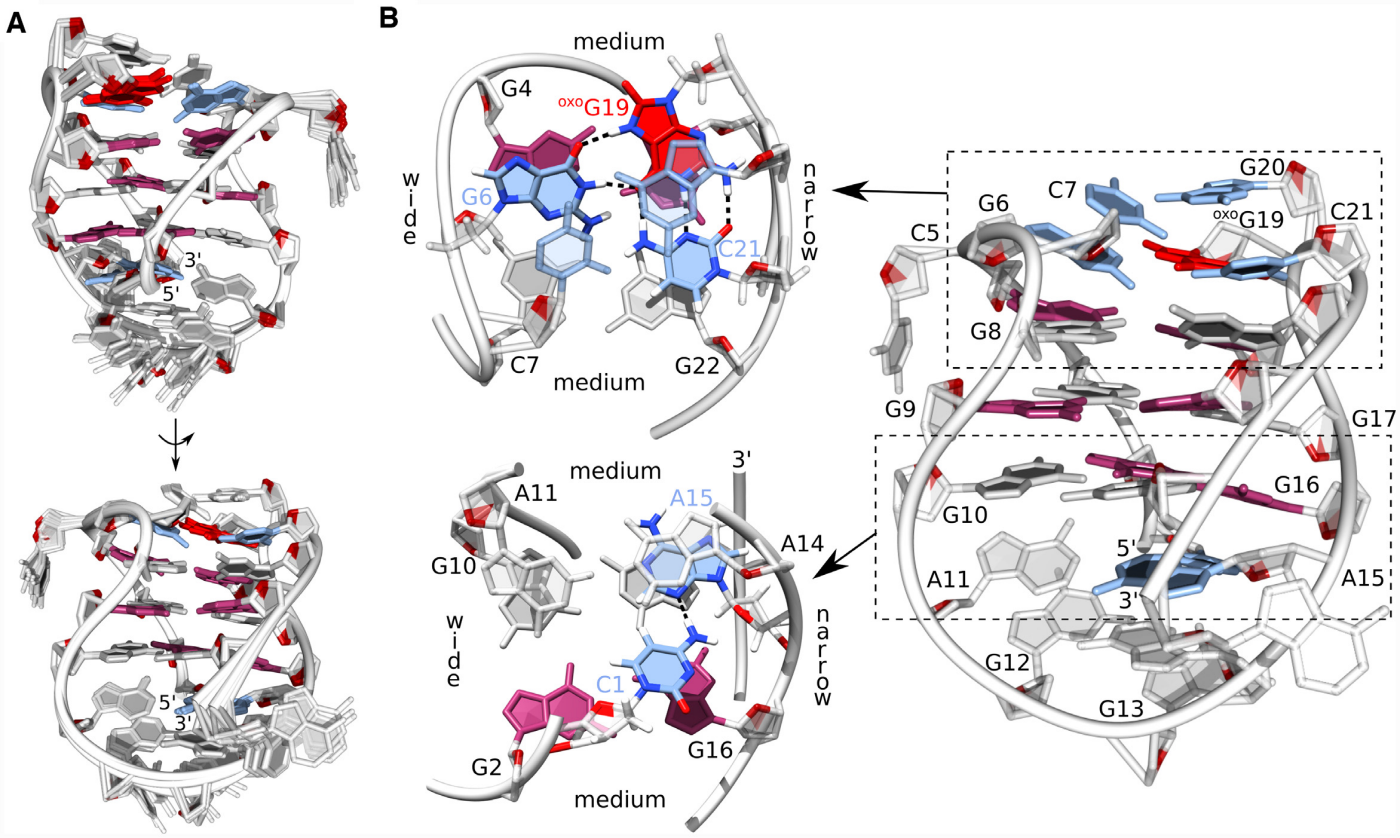
Structure of the bcl2ex-^oxo^G19 G-quadruplex. (**A**) Superpositions of 12 lowest energy structures presented in two views rotated by 180°. (**B**) Details of the representative structure. *Syn* and *anti* guanines are shown in magenta and gray, respectively, ^oxo^G is in red and base pairs/triad in blue.

A solution-state structure of the G-quadruplex adopted by bcl2ex-^oxo^G19 was solved using NMR data and a restrained simulated annealing protocol. A good convergence of structures was achieved with on average more than 12 NOE distance restraints per nucleotide (Table 1, Figure 5 and Supplementary Figure S10). The bcl2ex-^oxo^G19 G-quadruplex exhibits a wide groove (∼20 Å) between G2–G4 and G8–G10 tracts, two medium grooves (∼15 Å) between G2–G4 and G16–G18, as well as between G8–G10 and G22–G24, and a narrow groove (∼9 Å) between G16–G18 and G22–G24 (Figure 5B). ^oxo^G19 is not involved in G-quartet formation. Instead, its Watson–Crick edge is base paired with C21, while its Hoogsteen edge, which includes the protonated N7, is base paired with G6 thus forming the G6·^oxo^G19·C21 base triad. It is noteworthy that ^oxo^G19 adopts the *syn* glycosidic conformation. Furthermore, DQF-COSY spectrum showed that the predominant sugar pucker of ^oxo^G19 is north (*C3'-endo*) whereas the remaining nucleotides adopt the south (*C2'-endo*) pucker. Interestingly, C5 is bulged out of the G-quadruplex core and positioned in the wide groove, which facilitates efficient stacking of the G6·^oxo^G19·C21 base triad with the adjacent G-quartet (Supplementary Figure S8). Furthermore, C7·G20 form a Watson–Crick base pair that stacks on the G6·^oxo^G19·C21 base triad (Figure 5B). On the opposite side of the G-quadruplex core, C1 and A15 were found to interact via the C1H42–A15N3 hydrogen bond.

**Figure 6.**
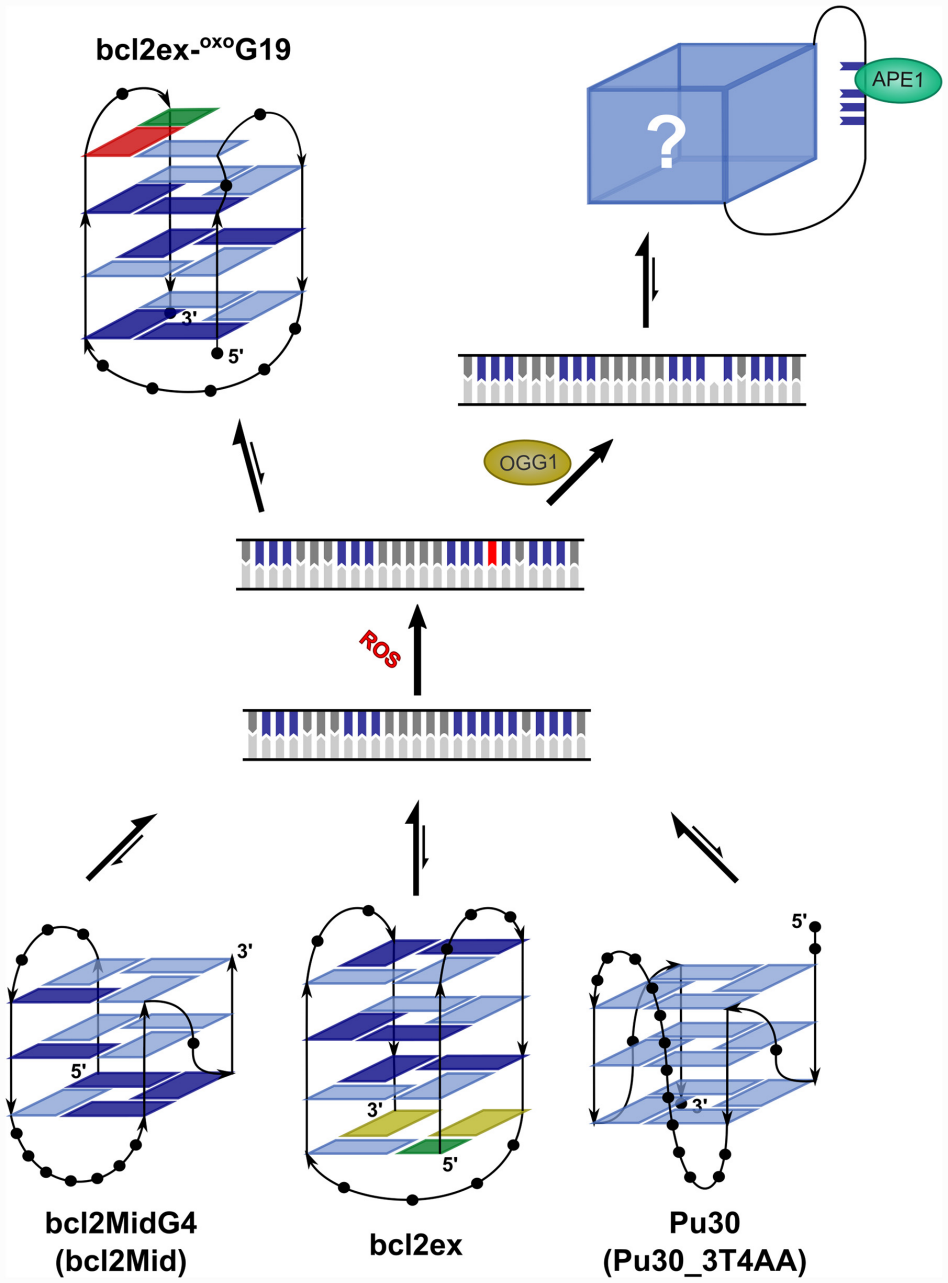
Proposed mechanism for oxidation of position G19 in the BCL2 promoter. Equilibrium between non-B-DNA structures, which include bcl2ex, is shifted towards the duplex form. In the event of oxidation ^oxo^G19 can be efficiently excised by OGG1, which destabilizes the duplex. The G-rich strand adopts an unknown G-quadruplex fold where the G_5_-tract is part of a long loop. Alternatively, the bcl2ex-^oxo^G19 G-quadruplex can form before ^oxo^G19 is excised. The oxidized G_5_-tract is involved in base pairing, which could hinder repair of the lesion.

Solvent accessibility and dynamics of individual G-quartets were evaluated with a deuterium for hydrogen exchange experiment (Supplementary Figure S11). Imino resonances of G17 and G23 in bcl2ex-^oxo^G19 could be observed even one month after transfer to ^2^H_2_O suggesting a tightly packed hydrophobic central cavity. On the other hand, imino protons of bcl2ex exchanged with deuterium within 12 h after H_2_O–^2^H_2_O transfer suggesting the G-quadruplex structure is more dynamic with a higher exposure of imino protons to solvent compared to bcl2ex-^oxo^G19.

## DISCUSSION

Promoters are double stranded regions of DNA, which can coexist in equilibrium with other secondary structures (e.g. G-quadruplex and i-motif). Non-B-DNA secondary structures within promoters are attractive targets for small molecule binding. Studies showed that stabilization of *BCL2* G-quadruplexes upon ligand binding inhibits *BCL2* expression and induces apoptosis, thus showing anti-cancer effects (38,39). G-quadruplex structures are notorious for their polymorphism that may involve variations in strand directionality, which results in either parallel, anti-parallel or hybrid topologies. Hurley *et al.* demonstrated that the G-rich strand of Pu39 is able to form different G-quadruplex structures with hybrid or predominantly parallel character (40). Many currently known G-quadruplexes in promoter regions adopt the parallel type of structure (9). Design of ligands for selective recognition of G-quadruplexes within a specific gene promoter is therefore challenging. However, parallel and antiparallel G-quadruplexes differ further in loop orientations and groove dimensions, which could be the basis for discrimination. The first oligonucleotide derived from the *BCL2* promoter, Bcl2MidG4, was studied in solution by Yang *et al.* (28). bcl2MidG4 contained the middle four G-tracts of Pu39, but was found to be structurally heterogeneous. Point mutations in the G_5_-tract were introduced (Bcl2Mid) to reduce structural polymorphism and solve a hybrid type G-quadruplex structure. Interestingly, we found that bcl2ex and bcl2MidG4 exhibit similar CD spectra (Supplementary Figure S2), which could suggest that a part of the ensemble of structures formed by bcl2MidG4 adopts ‘bcl2ex-like’ folds. Later, the longer Pu30 (first 30 nucleotides of Pu39) was found to adopt a parallel G-quadruplex structure with a 13-nt double-chain-reversal loop (41). The parent Pu30 again formed multiple structures in solution and mutations in the G5-tract and preceding G-tract (Pu30_3T4AA) were utilized for structure determination. While both bcl2Mid and Pu30_3T4AA folds exhibited comparable thermodynamic stabilities, the G-quadruplex with shorter loop lengths (i.e. bcl2Mid) was speculated to be kinetically favoured.

bcl2ex also contains the middle four Pu39’s G-tracts of which three are of equal length (GGG) while a single longer G_5_-tract is a potential source of structural polymorphism. The G-tract regions with connecting loops are formally extended in 5′ and 3′ directions by a single nucleotide (C1 and A25). These play an instrumental role in determination of the G-quadruplex structure of bcl2ex by NMR. C1 forms a Watson–Crick base pair with G16 and both are adjoined by A11 and A25 to form a planar CGAA-quartet. This structural element apparently stabilizes only a single basket-type G-quadruplex as no strand slippage derived polymorphism or other competing folds were observed. Interestingly, contribution of the CGAA-quartet to the overall thermal stability of the bcl2ex G-quadruplex is negligible since its melting temperature is comparable to that of bcl2MidG4 (69°C). An unusual structural feature of the CGAA-quartet is the *syn* conformation of C1. Antiparallel and hybrid type G-quadruplexes often start with a 5′ guanine in *syn* conformation (42–46). However, in bcl2ex the G-quadruplex core starts with G2 in the *anti* conformation and is followed by a *syn* G3, a sequence step that was shown to be generally unfavourable by free energy calculations (47). C1 in *syn* conformation could therefore facilitate formation of the unfavourable *anti–syn* step in the G-quadruplex core.

The Xodo laboratory found that DNA oxidation can upregulate KRAS gene expression (21). Facilitated by DNA supercoiling the G-rich region within KRAS can form G-quadruplex structures. Upon oxidation the G-quadruplex fold is retained if ^oxo^G appears in long loop regions. However, ^oxo^G increases binding of transcription factors to the G-quadruplex, which destabilizes the structure. DNA reverts to the duplex form and ^oxo^G can be efficiently repaired by base excision repair (BER) where the nucleobase is excised by the ^oxo^G glycosylase 1 (OGG1). This creates an abasic site (AP), which is cleaved by the apurinic/apyrimidinic endonuclease 1 (APE1). Lesion free promoter DNA has increased affinity towards nuclear factors, which activates KRAS transcription. Regulation of *BCL2* expression, which sensitizes cells to apoptosis, was also shown to be related to exposure to ROS (48–51). A report by Avvedimento *et. al*. revealed an intricate mechanism of targeted ^oxo^G formation in the *BCL2* promoter, which activates gene expression (52). Results of the current study complement earlier observations by showing that oxidation of certain guanines within the *BCL2* promoter could regulate *BCL2* expression by shifting equilibrium between duplex and G-quadruplex structures. Substitution of G19 to ^oxo^G formally splits the G_5_-tract into a three guanine tract and an isolated guanine, which has an extensive effect on the 3D structure. Comparison of bcl2ex and bcl2ex-^oxo^G19 structures shows that G19 shifts register and switches position from constituting the top G-quartet in bcl2ex to the G6·^oxo^G19·C21 base triad in bcl2ex-^oxo^G19. Hoogsteen type hydrogen bonding such as that of an *anti* G6 and a *syn*^oxo^G19 has been observed previously and shown as energetically favourable (53). On the other hand, Watson–Crick hydrogen bonding between ^oxo^G19 and C21 is unusual as both nucleotides are in the *syn* conformation. Due to the G_5_-tract changing register, G16 is no longer available for base pairing with C1, which precludes the formation of the CGAA-quartet in bcl2ex-^oxo^G19. It is noteworthy that comparable G-quartets in bcl2ex and bcl2ex-^oxo^G19 differ in hydrogen bond directionalities, which suggests that the switch to the bcl2ex-^oxo^G19 structure would have to involve an unfolded or double helical intermediate. Interestingly, both bcl2ex and bcl2ex-^oxo^G19 exhibit capping structures flanking their G-quadruplex cores, which is in agreement with previously observed basket-type G-quadruplexes in solutions containing K^+^ ions where extensive base pairing and stacking was found in the loops (54,55).

We have recently shown that the structure of a hybrid type human telomeric G-quadruplex is disrupted when ^oxo^G is substituted in the central G-quartet, while the effect is less severe when substitutions are made in the outer G-quartets (24). The human telomeric G-quadruplex can tolerate oxidation of certain guanines, which are in *syn* conformation. On the other hand, if a guanine in *anti* conformation is affected, a major conformational shift is required to accommodate the lesion. A similar trend was observed in a study by Myong *et. al*. where the central G-quartet position of a human telomeric G-quadruplex was the most destabilizing when substituted by ^oxo^G (56). However, direct oxidation of guanines in the central G-quartet is unlikely, because they are protected by the surrounding G-quadruplex structure (57). Capping structures of the bcl2ex G-quadruplex could offer an even higher degree of protection against direct oxidation. G20 is redundant in the G_5_-tract and is exposed to the solvent in the bcl2ex structure. Interestingly, virtually no change in G-quadruplex structure or stability is observed when G20 in bcl2ex is substituted with ^oxo^G. On the other hand, G19 is involved in G-quartet formation and is highly unlikely to be oxidized while the bcl2ex G-quadruplex is folded.

The G-rich strand of the BCL2 promoter exists in equilibrium between duplex and G-quadruplex forms. This equilibrium is likely shifted towards the duplex form where G-tracts are likely targets for oxidation, either direct or via electron hole migration (58). Due to the number of successive Gs the G_5_-tract in bcl2ex should be highly susceptible to oxidation. However, based on data for VEGF and NEIL3 systems (29,30), other G-tracts in bcl2ex are equally likely to be oxidized. Computational studies showed that within a G-tract the 3′ G, especially when followed by a C, is the most protected from oxidation (59). Analogously, in the G_5_-tract of bcl2ex, G20 should be protected, while the four upstream Gs are likely candidates for oxidation. However, the BCL2 promoter (beyond bcl2ex) contains additional G-tracts of different lengths, which offers a level of redundancy. Burrows *et al.* have shown that redundant G-tracts in VEGF and NEIL3 promoters play a pivotal role in regulating gene expression (22,30). Oxidative lesions were found to upregulate gene expression by destabilizing the duplex and shifting the structural equilibrium in favour of G-quadruplexes. ^oxo^G in duplex DNA is normally removed by BER. This creates an AP site, which is highly destabilizing to the duplex and thus promotes formation of a G-quadruplex structure. The AP site is excluded from the G-quadruplex core by utilizing redundant G-tracts. Interestingly, AP sites were suggested to decreases the number of possible structures and slightly stabilizes the fold. Subsequently, APE1 is recruited to the excluded AP sites, but does not cleave the DNA strand. Instead, APE1 recruits transcription factors, which generally upregulate gene expression. However, depending on location, strand, and promoter the reverse effect of gene suppression can also be observed and may involve i-motif structures (60). The same mechanism could also regulate BCL2 expression where upon oxidation structural equilibrium would shift from a double helical towards the G-quadruplex form (Figure 6). Normally this would include formation of an AP site, which would destabilize the duplex. However, oxidation in the bcl2ex region at position G19 of the double helix could shift the equilibrium in favour of G-quadruplex before removal of ^oxo^G19 with APE1. While ^oxo^G19 is not expected to cause a major decrease in duplex stability, the change in structural equilibrium would be driven by increased stability of the oxidized G-quadruplex. Furthermore, once the bcl2ex-^oxo^G19 structure is formed, repair could be less efficient due to high thermal stability of the G-quadruplex. OGG1 initiates excision by hydrogen bonding with the protonated N7 of ^oxo^G (61,62). In the structure adopted by bcl2ex-^oxo^G19, H7 of ^oxo^G19 is hydrogen bonded to G6, which could negatively affect removal of ^oxo^G19 by OGG1. Additional structural transformations to partially or completely unfold the structure could be required. On the other hand, the structure of bcl2ex-^oxo^G19 could hinder ROS from accessing ^oxo^G19 and prevent secondary oxidation reactions despite the low redox potential of ^oxo^G. The inability to remove ^oxo^G19 could lead to a stable lesion, which would act as an epigenetic modification that might regulate gene expression by altering the binding of transcription factors to the DNA.

In summary, identification of new non-B-DNA secondary structures adds additional levels of complexity to the folding landscape of the G-rich region of the P1 promoter of *BCL2* and suggests that multiple structures are relevant for regulation of transcription. Diversity of *BCL2* G-quadruplex structures is further expanded by the action of reactive oxygen species. While oxidative lesions were mostly found to reduce G-quadruplex stability, we report a significant stabilizing effect on a biologically relevant G-quadruplex. Unique structural features presented in this study could prove significant for detection and mitigation of oxidative lesions in the human BCL2 gene and related diseases.

## DATA AVAILABILITY

The coordinates for the 12 lowest energy structures of bcl2ex and bcl2ex-oxoG19 have been deposited in the Protein Data Bank with accession codes 6ZX7 and 6ZX6, respectively. Chemical shifts have been deposited in the Biological Magnetic Resonance Data Bank as entries 34543 and 34542.

## Supplementary Material

gkab057_Supplemental_File
