# Supplementary material for: Oxidative lesions modulate G-quadruplex stability and structure in the human BCL2 promoter
Source: Nucleic Acids Res. 2021 Feb 8;49(4):2346–56. doi: 10.1093/nar/gkab057 (PMC7913773; doi:10.1093/nar/gkab057)
Supplement: gkab057_Supplemental_File [file gkab057_supplemental_file.pdf]

SUPPORTING INFORMATION

Oxidative lesions modulate G-quadruplex stability and structure in the human BCL2 promoter

Stasė Bielskutė[1], Janez Plavec[1,2,3] and Peter Podbevšek[1]

[1]Slovenian NMR Center, National Institute of Chemistry, Hajdrihova 19, SI-1000 Ljubljana, Slovenia

[2]EN-FIST Center of Excellence, Trg OF 13, SI-1000 Ljubljana, Slovenia

[3]Faculty of Chemistry and Chemical Technology, University of Ljubljana, Večna pot 113, SI-1000 Ljubljana, Slovenia

List of Figures

Figure S1. UV melting curves of bcl2ex and its analogues with oxoG substitutions in the G5-tract...... 2
Figure S2. CD spectra of bcl2ex, bcl2ex-^{oxo}G19 and bcl2MidG4. 2
Figure S3. 1D ^{15}N-edited HSQC spectra of bcl2exT and bcl2ex.. 3
Figure S4. bcl2ex and bcl2exT: comparisons of oligonucleotides' sequences, ^{1}H, NOESY and ^{13}C HSQC NMR spectra.. 4
Figure S5. ^{13}C HSQC spectra of bcl2ex and bcl2ex-^{oxo}G19. 5
Figure S6. bcl2ex constructs with 5' extensions. 6
Figure S7. NOE distance restraints for bcl2ex. 7
Figure S8. Comparison of stacking between different layers in bcl2ex and bcl2ex-^{oxo}G19. 8
Figure S9. 1D ^{15}N-edited HSQC NMR spectra of bcl2ex-^{oxo}G19 oligonucleotides. 9
Figure S10. NOE distance restraints for bcl2ex-^{oxo}G19... 10
Figure S11. Deuterium exchange experiments for bcl2ex and bcl2ex-^{oxo}G19.......................................11

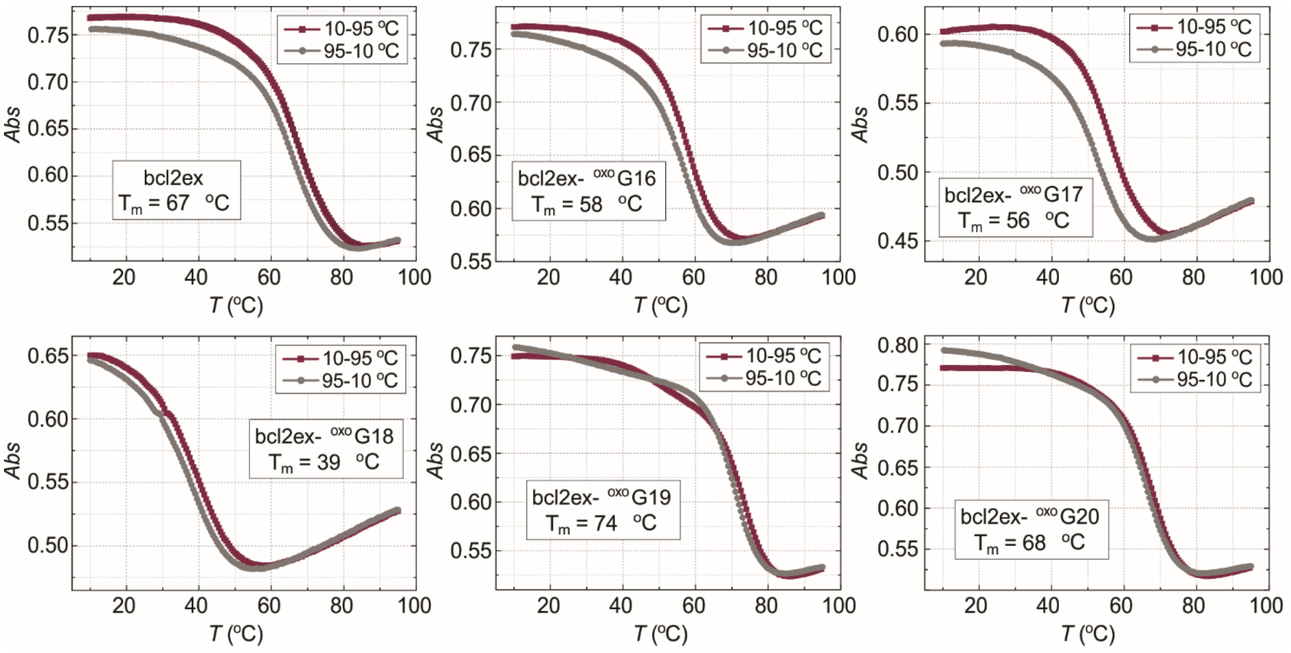

Figure S1. UV melting curves of bcl2ex and its analogues with ^{oxo}G substitutions in the G_5-tract. Data were acquired for DNA with 70 mM KCl and 20 mM K-phosphate buffer, pH 7, oligonucleotide concentrations were ~10 μM. Thermal melting analysis was initiated by equilibrating samples at 10 °C for 10 min before being heated to 95 °C (magenta curve) then after 10 min temperature was reduced to 10 °C (grey curve) at a rate of 0.5 °C/min. Absorbance was followed at 295 nm.

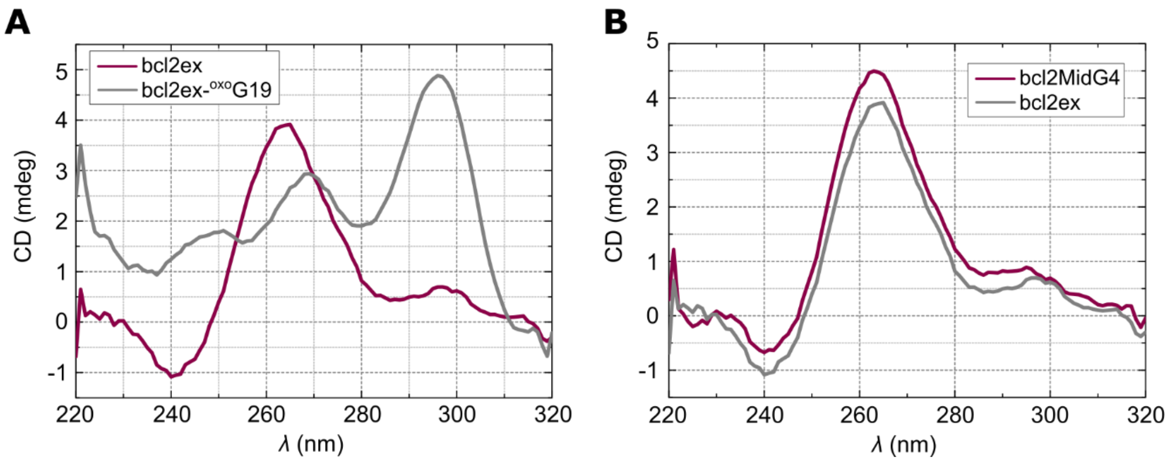

Figure S2. Comparison of CD spectrum of bcl2ex with (A) bcl2ex-^{oxo}G19 and (B) bcl2MidG4. Data were acquired for DNA with 70 mM KCl and 20 mM K-phosphate buffer, pH 7, oligonucleotide concentrations were 50 μM.

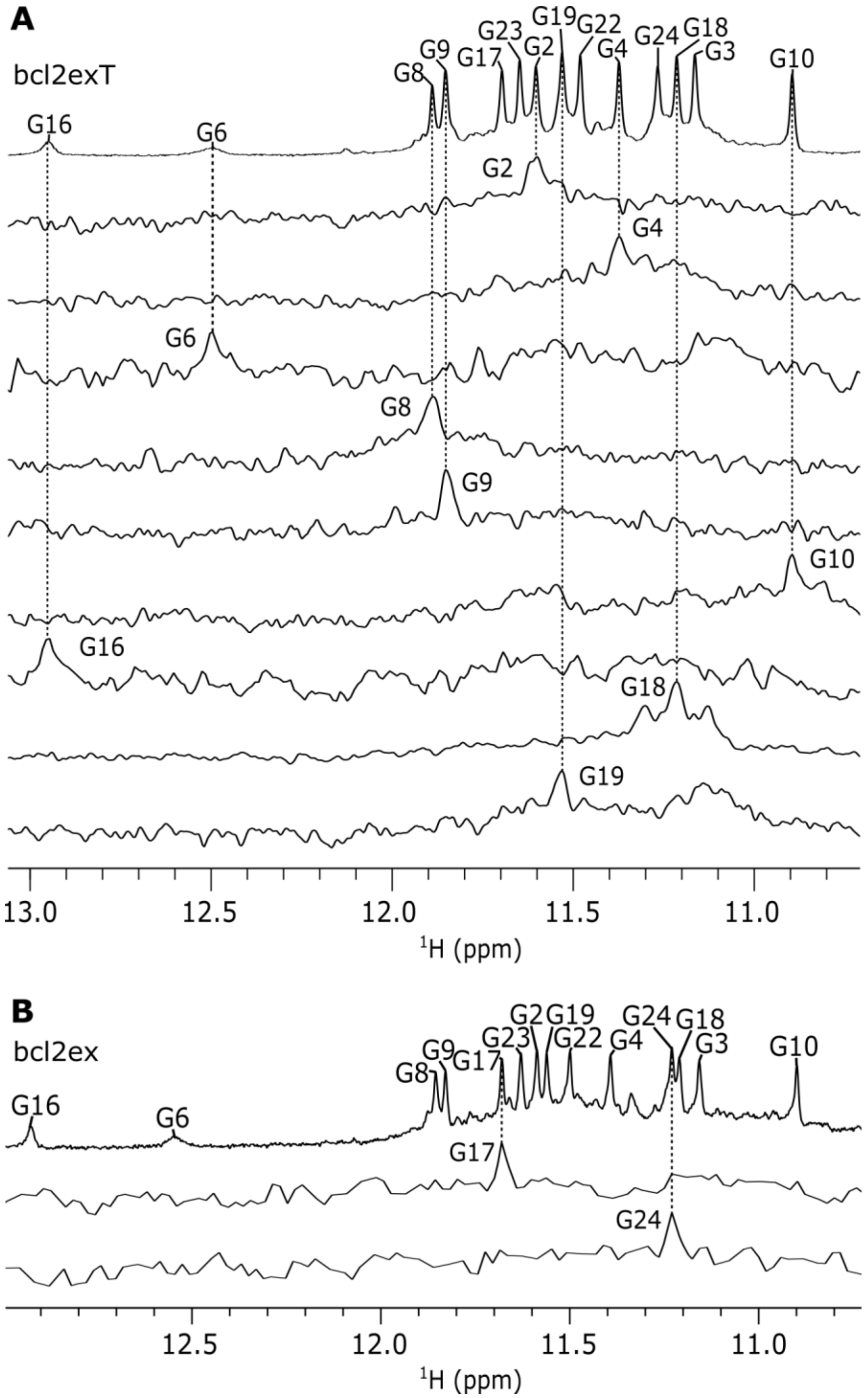

Figure S3. Imino regions of 1D ^{15}N-edited HSQC spectra of site specifically ^{15}N labeled (A) bcl2exT and (B) bcl2ex. Resonance assignments are shown on the top of spectra. Spectra were acquired in 90% H_2O and 10% ^2H_2O, 70 mM KCl, and 20 mM K-phosphate buffer, pH 7 at 25 °C, on a 600 MHz NMR spectrometer. Oligonucleotide concentrations were ~0.5 mM.

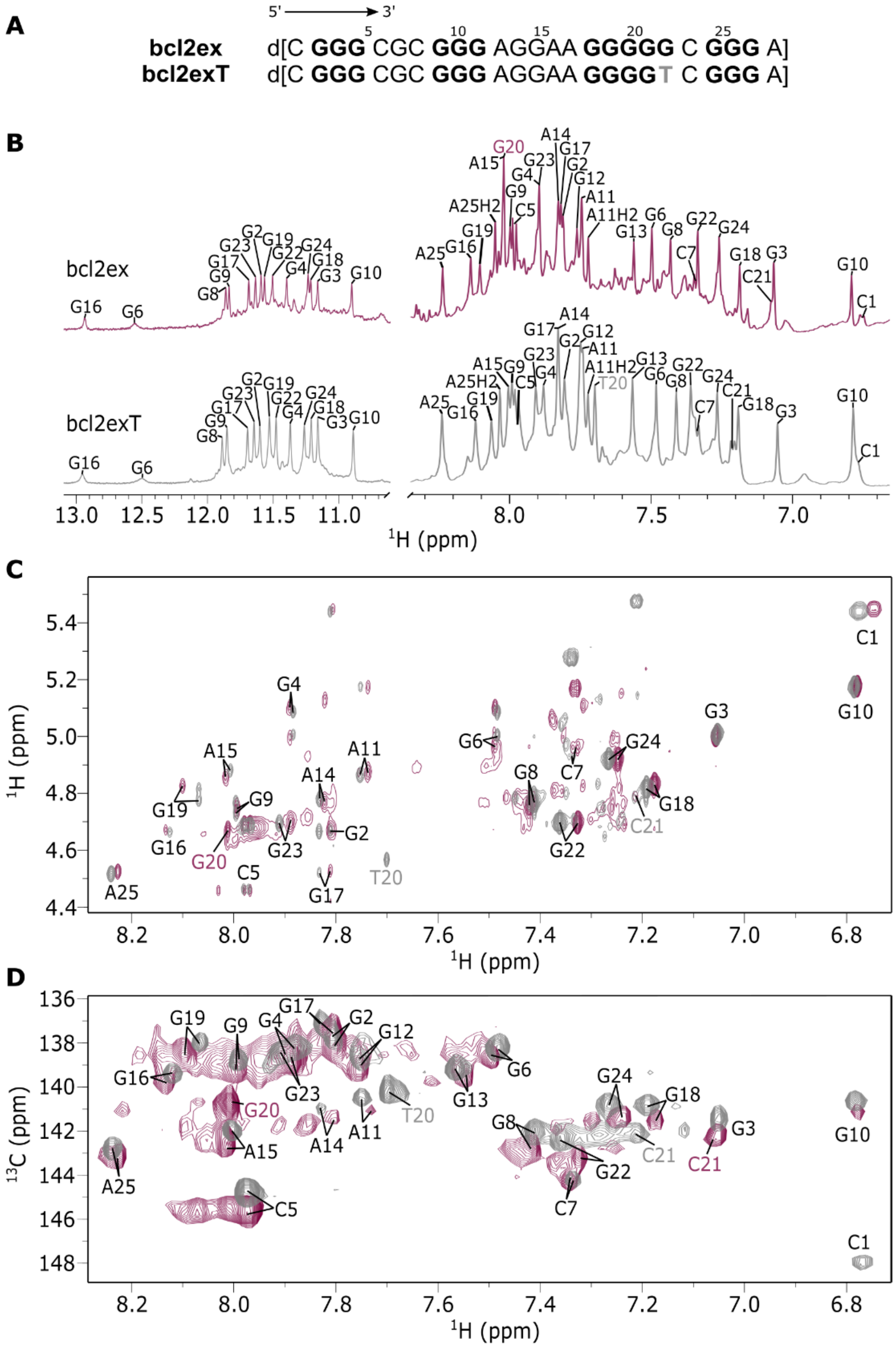

Figure S4. Bcl2ex and bcl2exT comparisons. (A) Oligonucleotides' sequences. T20 substitution is shown in magenta. (B) ^{1}H NMR spectra of imino and aromatic regions. Assignments are shown on the top of spectra. (C) NOESY (τ_m = 300 ms and τ_m = 350 ms, respectively) spectra of aromatic-anomeric regions. Assignments are shown next to intranucleotide H6/H8-H1' cross-peaks. (D) ^{1}H-^{13}C HSQC spectra showing H8/H6-C8/C6 correlations. Bcl2ex and bcl2exT spectra are in magenta and grey, respectively.

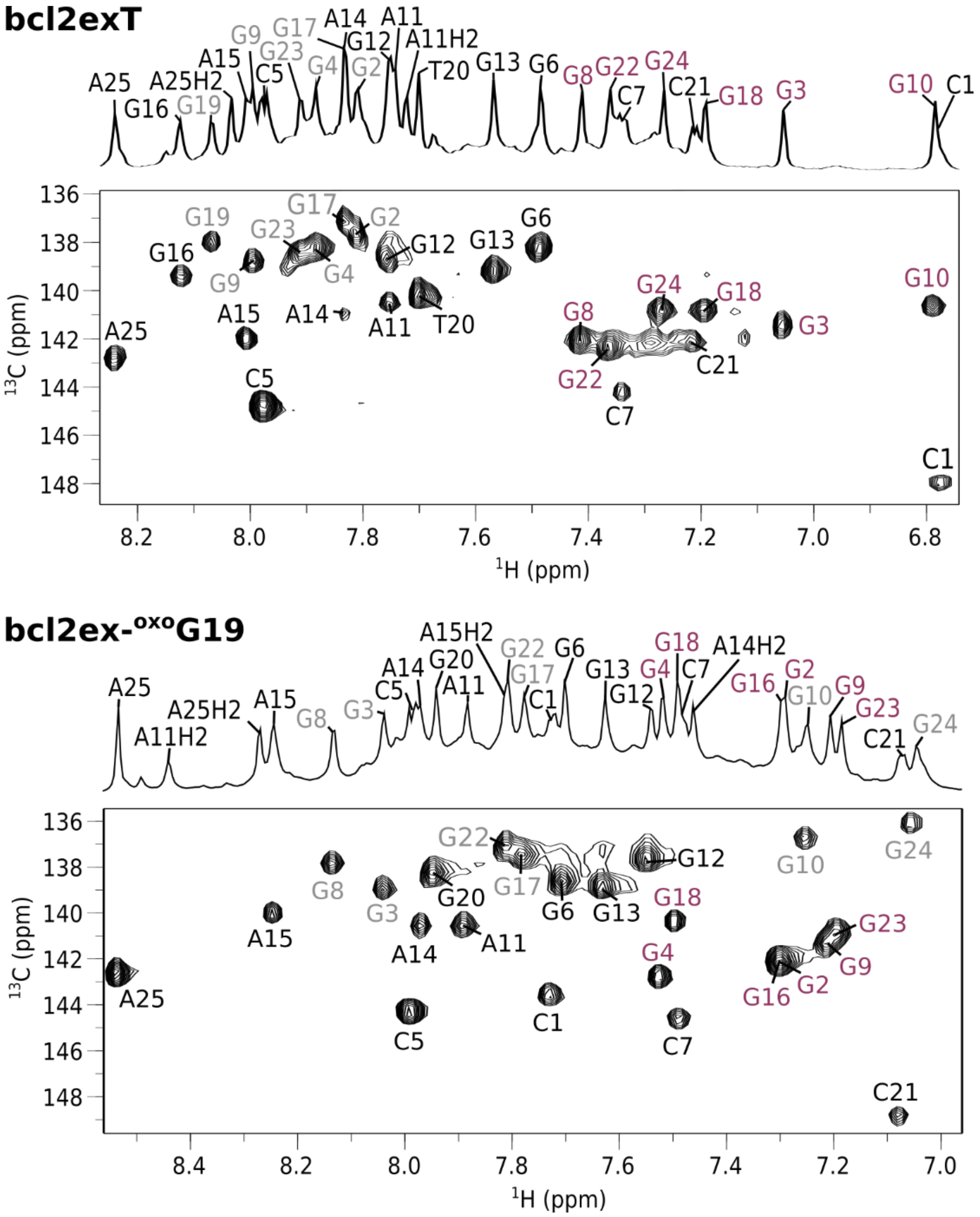

Figure S5. Aromatic regions of ^{13}C HSQC NMR spectra of bcl2exT and bcl2ex-^{oxo}G19. Spectra were acquired in 100% ^2H_2O, 70 mM KCl, and 20 mM K-phosphate buffer, pH 7 at 25 °C, on a 600 MHz NMR spectrometer.

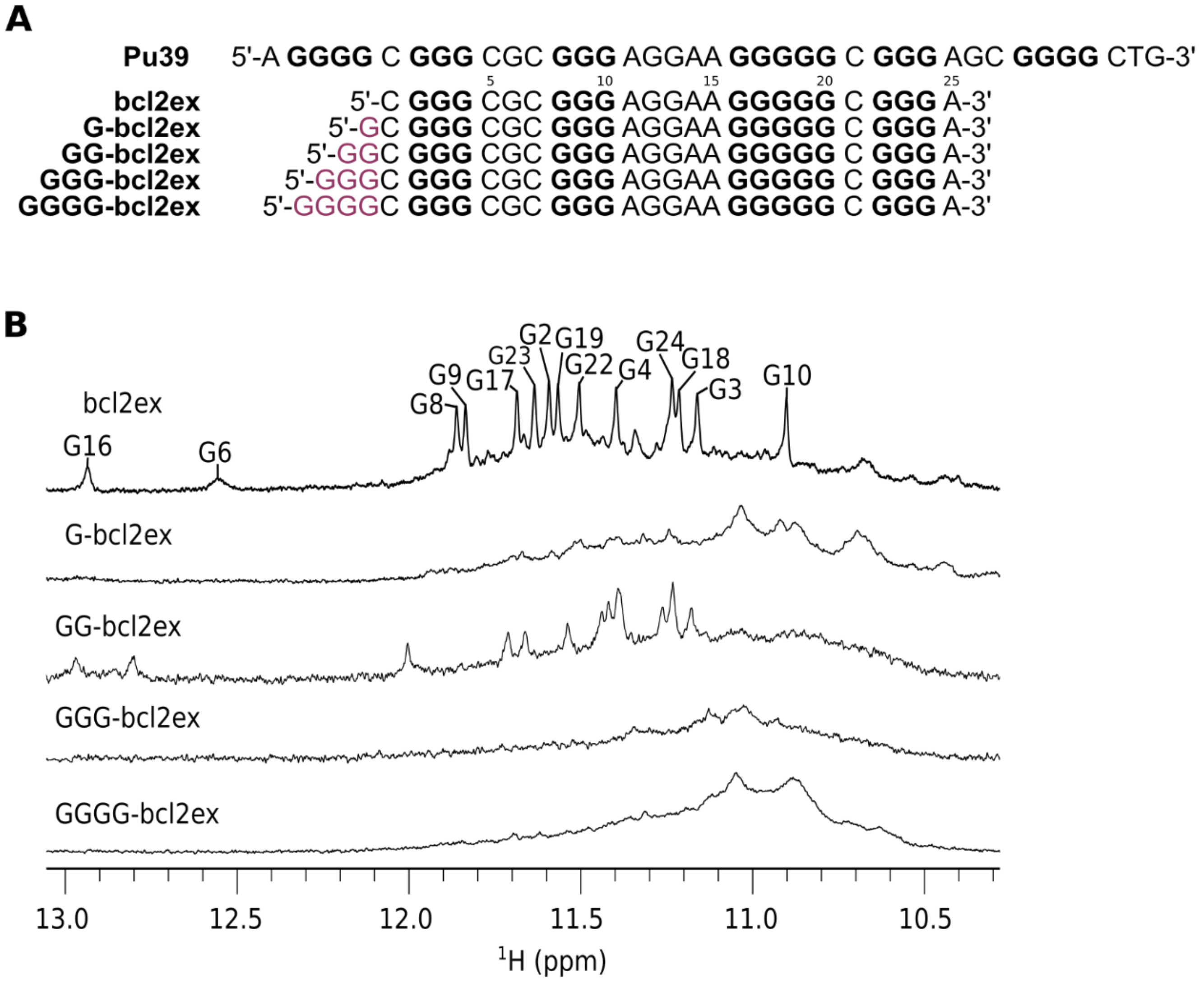

Figure S6. (A) Oligonucleotide sequences of bcl2ex constructs with 5’ extensions. (B) Imino regions of their 1D ^{1}H NMR spectra acquired in 90% H_2O and 10% ^2H_2O, 70 mM KCl, and 20 mM K-phosphate buffer, pH 7 at 25 °C, on a 600 MHz NMR spectrometer..

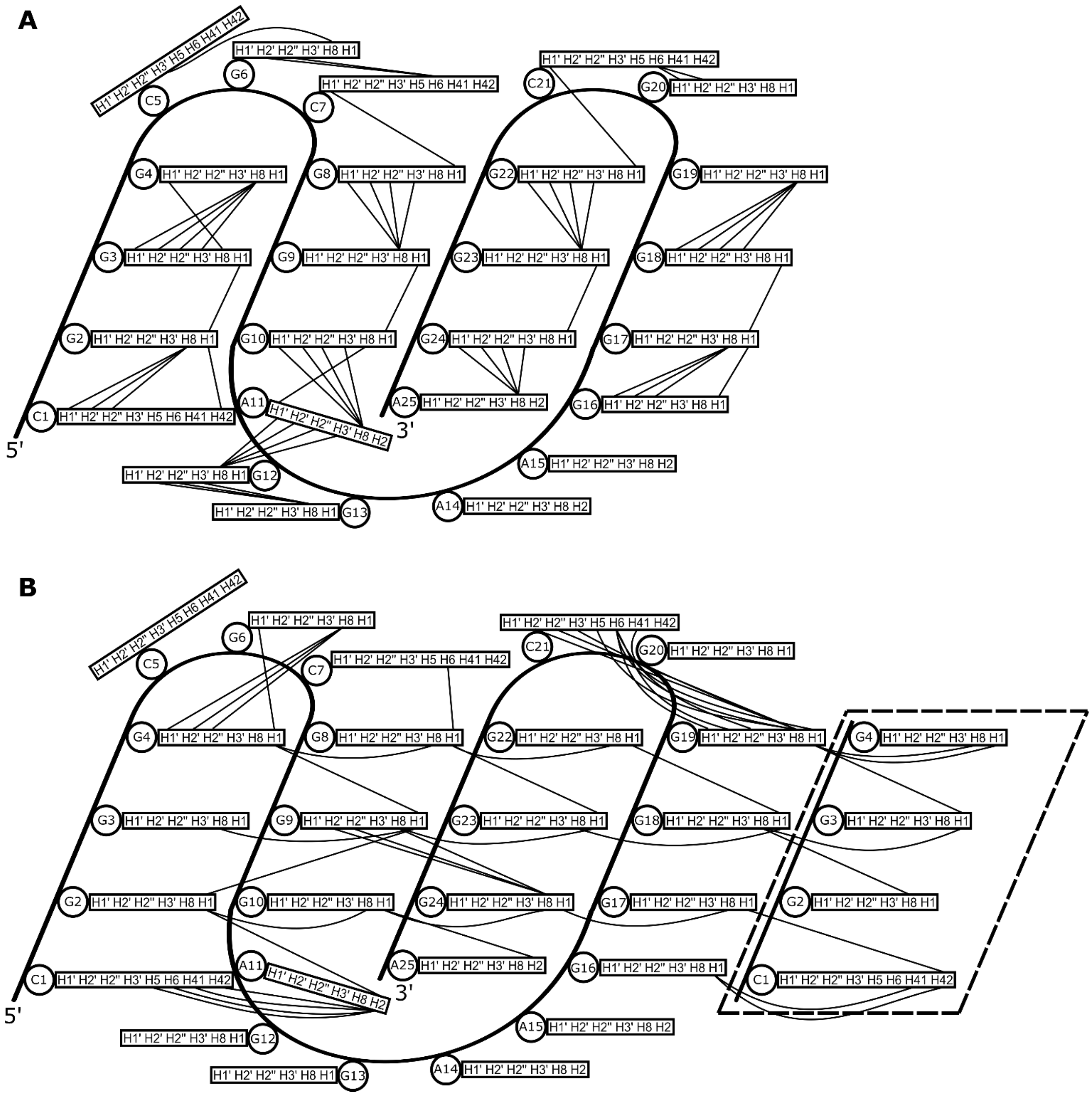

Figure S7. NOE distance restraints used in structure determination of bcl2ex. (A) Short- and (B) long-range restraints are shown as lines between individual atoms of nucleotides.

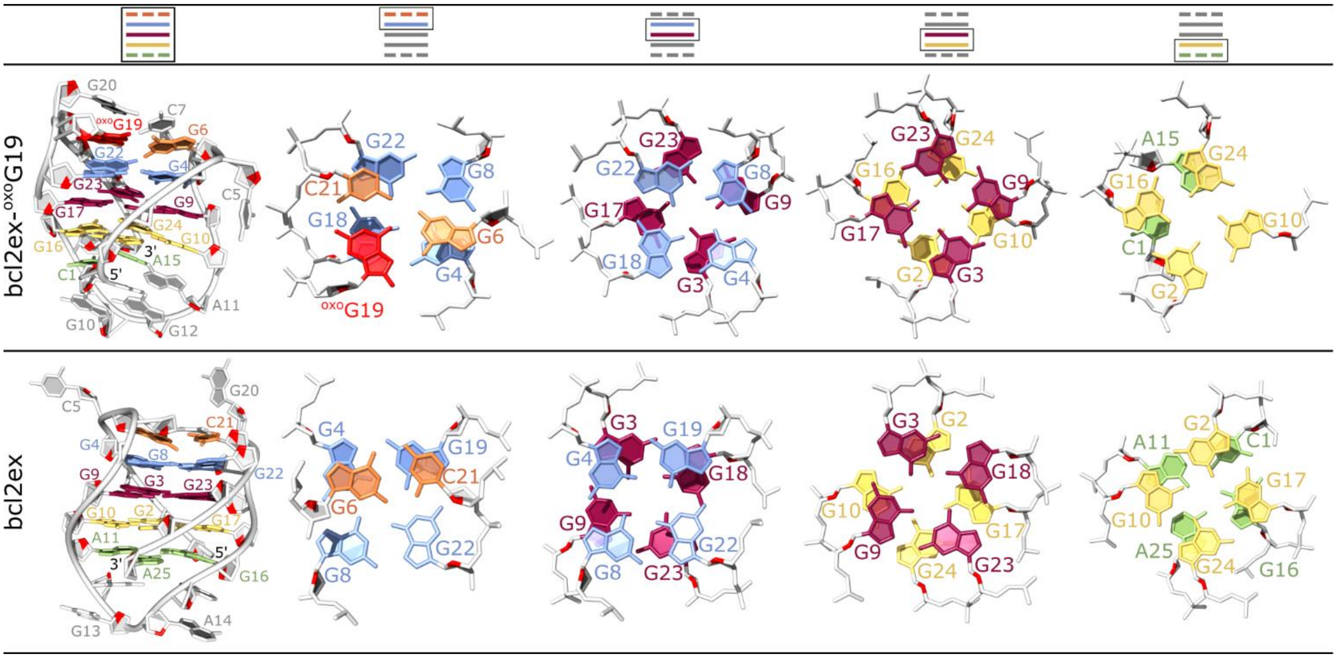

Figure S8. Comparison of stacking between different layers in bcl2ex-^{oxo}G19 and bcl2ex. Bottom, central and top G-quartets are in yellow, magenta and blue, respectively. Stacked loop nucleotides are in orange and grey. ^{oxo}G is shown in red.

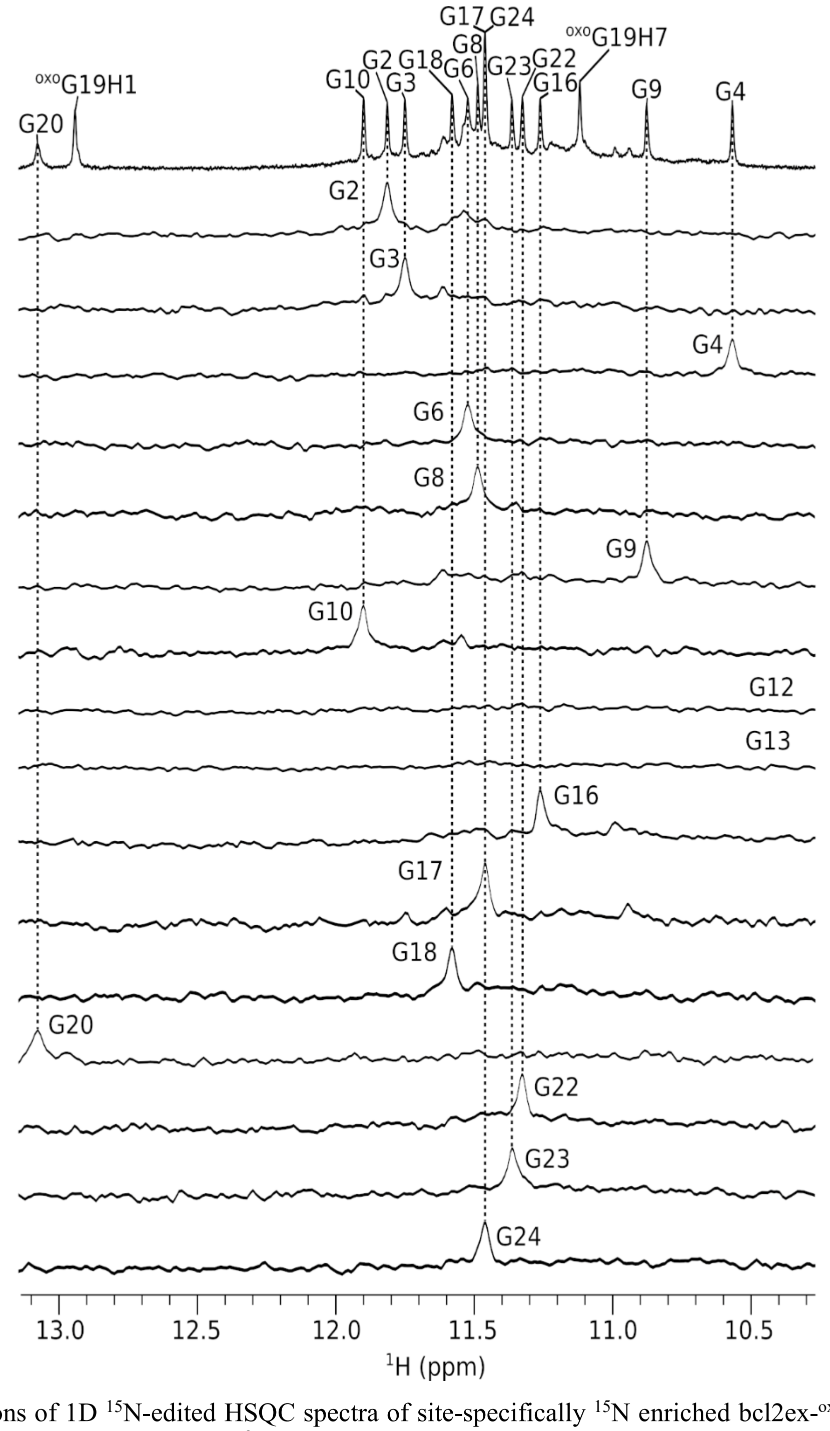

Figure S9. Imino regions of 1D ^{15}N-edited HSQC spectra of site-specifically ^{15}N enriched bcl2ex-$^{oxo}G19$ oligonucleotides. Spectra were acquired in 90% H_2O and 10% ^2H_2O, 70 mM KCl, and 20 mM K-phosphate buffer, pH 7 at 25 °C, on a 600 MHz NMR spectrometer. Oligonucleotide concentrations were ~0.5 mM.

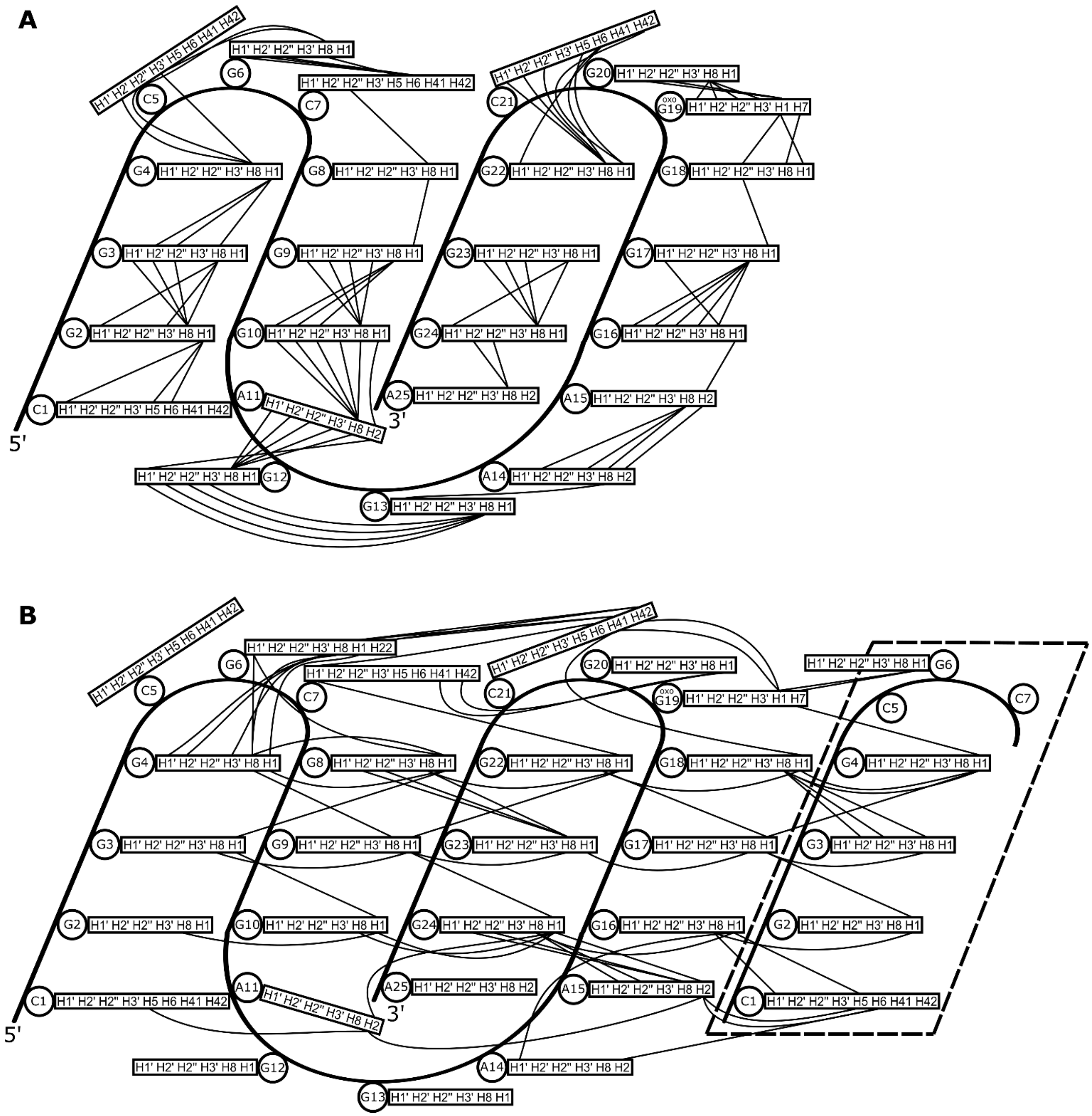

Figure S10. NOE distance restraints used in structure determination of bcl2ex-^{oxo}G19. (A) Short- and (B) long-range restraints are shown as lines between individual atoms of nucleotides.

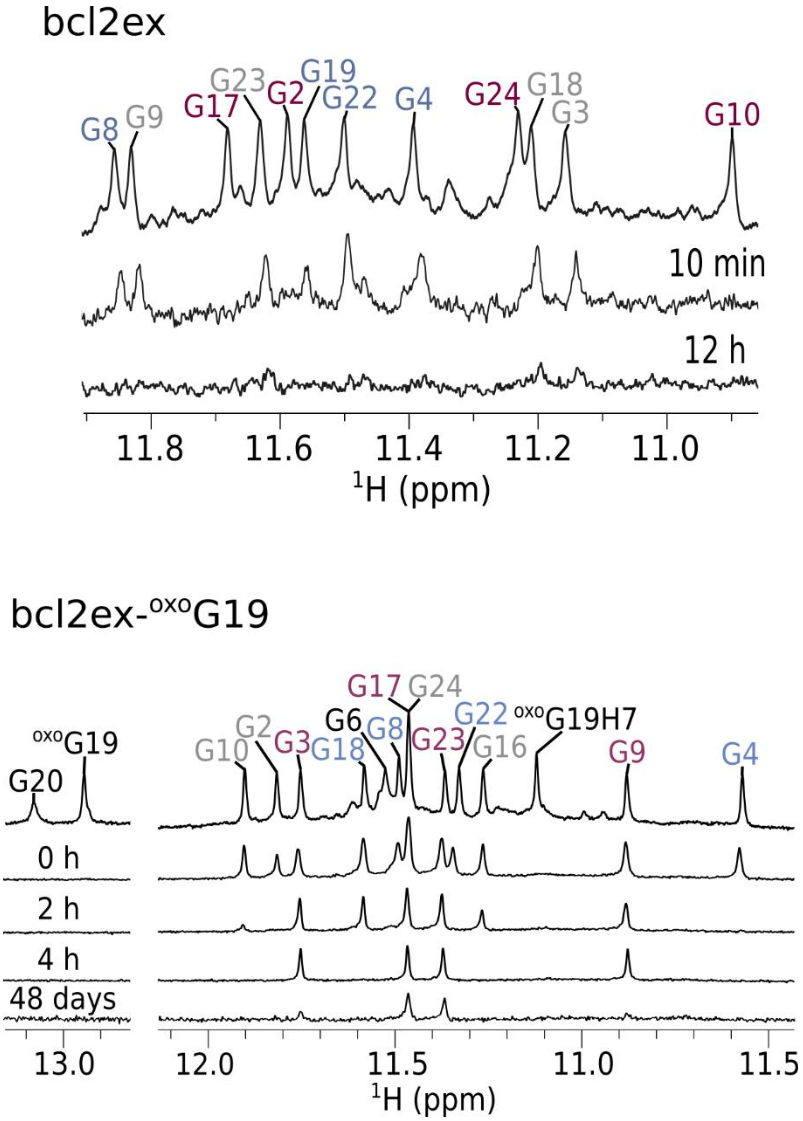

Figure S11. Imino regions of 1D ^1H spectra of bcl2ex and bcl2ex-^{oxo}G19 in 10% ^2H_2O and changes after transfer to 100% ^2H_2O. Spectra were acquired in 70 mM KCl, and 20 mM K-phosphate buffer, pH 7 at 25 °C, on a 600 MHz NMR spectrometer. Oligonucleotide concentrations were ~0.7 mM.
